# Effect of early vs. delayed extubation on functional outcome among patients with acute ischemic stroke treated with endovascular thrombectomy under general anesthesia: the prospective, randomized controlled EDESTROKE trial study protocol

**DOI:** 10.1186/s13063-024-08181-y

**Published:** 2024-06-04

**Authors:** Manuel Taboada, Ana Estany-Gestal, Jorge Fernández, Laura Barreiro, Kora Williams, Manuel Rodríguez-Yáñez, Pablo Otero, Alberto Naveira, Valentín Caruezo, Sonia Veiras, Eva San Luis, Laura Dos Santos, María Diaz-Vieito, Susana Arias-Rivas, María Santamaría-Cadavid, Emilio Rodríguez-Castro, Fernando Vázquez, Miguel Blanco, Antonio Mosquera, Jose Antonio Castiñeiras, Ignacio Muniategui, Esteban Ferreiroa, Agustín Cariñena, Ana Tubio, Olga Campaña, Salomé Selas, Francisco Aneiros, Adrián Martínez, María Eiras, Jose Costa, Jose María Prieto, Julián Álvarez

**Affiliations:** 1 Department of Anesthesiology, University Clinical Hospital of Santiago, Santiago, Spain; 2 Research Methodology Unit, Fundación Instituto de Investigaciones Sanitarias (FIDIS), Santiago, Spain; 3 Department of Neurology, University Clinical Hospital of Santiago, Santiago, Spain; 4 Department of Neuroradiology, University Clinical Hospital of Santiago, Santiago, Spain

**Keywords:** Stroke, Endovascular treatment, Extubation, Mechanical ventilation, Functional status

## Abstract

**Background:**

Recent meta-analyses and randomized studies have shown that among patients with acute ischemic stroke undergoing endovascular thrombectomy, general anesthesia with mechanical ventilation is associated with better functional status compared to local anesthesia and sedation, and they recommend its use. But once the procedure is completed, when is the optimal moment for extubation? Currently, there are no guidelines recommending the optimal moment for extubation. Prolonged mechanical ventilation time could potentially be linked to increased complications such as pneumonia or disturbances in cerebral blood flow due to the vasodilatation produced by most anesthetic drugs. However, premature extubation in a patient who has suffered a stroke could led to complications such as agitation, disorientation, abolished reflexes, sudden fluctuations in blood pressure, alterations in cerebral blood flow, respiratory distress, bronchial aspiration, and the need for reintubation. We therefore designed a randomized study hypothesizing that early compared with delayed extubation is associated with a better functional outcome 3 months after endovascular thrombectomy treatment under general anesthesia for acute ischemic stroke.

**Methods:**

This investigator-initiated, single-center, prospective, parallel, evaluated blinded, superiority, randomized controlled trial will include 178 patients with a proximal occlusion of the anterior circulation treated with successful endovascular thrombectomy (TICI 2b-3) under general anesthesia. Patients will be randomly allocated to receive early (< 6 h) or delayed (6–12 h) extubation after the procedure. The primary outcome measure is functional independence (mRS of 0–2) at 90 days, measured with the modified Rankin Score (mRS), ranging from 0 (no symptoms) to 6 (death).

**Discussion:**

This will be the first trial to compare the effect of mechanical ventilation duration (early vs delayed extubation) after satisfactory endovascular thrombectomy for acute ischemic stroke under general anesthesia.

**Trial registration:**

The study protocol was approved April 11, 2023, by the by the Santiago-Lugo Research Ethics Committee (CEI-SL), number 2023/127, and was registered into the clinicaltrials.gov clinical trials registry with No. NCT05847309. Informed consent is required. Participant recruitment begins on April 18, 2023. The results will be submitted for publication in a peer-reviewed journal and presented at one or more scientific conferences.

**Supplementary Information:**

The online version contains supplementary material available at 10.1186/s13063-024-08181-y.

## Introduction

### Background and rationale {6a}

Endovascular thrombectomy in addition to the medical treatment is, in this moment, the standard of care for patients who had a stroke caused by a large vessel occlusion in the anterior circulation [1–6]. Several factors have been described that may influence the evolution and functional status at 3 months of patients who have suffered a stroke and have received endovascular thrombectomy, such as the time between the onset of symptoms and admission to the ward for performing the procedure, adequate control of blood pressure, the size of the cerebral infarct, a worse neurological examination at the time of the procedure, or the type of anesthesia (sedation versus general anesthesia).

Until a few years ago, sedation was recommended over general anesthesia during endovascular thrombectomy for acute ischemic stroke. Recent meta-analyses and randomized studies have shown that general anesthesia with mechanical ventilation is associated with better functional status at 3 months compared to local anesthesia and sedation [7–15], and they recommend its use. But once the procedure is completed, when is the optimal moment of extubation? Currently, there are no guidelines recommending the optimal moment for extubation after endovascular thrombectomy of the patient with stroke under general anesthesia. Prolonged mechanical ventilation time could potentially be linked to increased complications such as pneumonia or disturbances in cerebral blood flow due to the vasodilatation produced by most anesthetic drugs. However, premature extubation in a patient who has suffered a stroke could led to complications such as agitation, disorientation, abolished reflexes, sudden fluctuations in blood pressure, alterations in cerebral blood flow, respiratory distress, bronchial aspiration, and the need for reintubation [16–19].

In two observational studies, authors observed that mechanical ventilation times exceeding 24 h were associated with a higher incidence of pneumonia and a worse functional state at 3 months. However, these are observational studies, and the timing of extubation may have been influenced by other factors such as the size and location of the infarct, the NIHSS (National Institutes of Health Stroke Scale), or the neurological status prior to endotracheal intubation. Therefore, it is challenging to establish a direct relationship between extubation timing and functional prognosis at 3 months in an observational study. Consequently, there is a need for a trial determining whether early extubation (< 6 h) compared to delayed extubation (6–12 h) can influence the evolution and functional status at 3 months of patients who have suffered a stroke and have received satisfactory endovascular thrombectomy under general anesthesia.

We therefore designed a randomized study hypothesizing that early compared with delayed extubation is associated with a better functional outcome, according to the modified Rankin scale, 90 days after endovascular thrombectomy under general anesthesia for acute ischemic stroke.

### Objectives {7}

#### Primary objective

The aim of this prospective randomized study is to compare the effect of early (< 6 h) versus delayed (6–12 h) extubation on functional outcome at 90 days, as measured by the modified Rankin scale (mRS), in patients with acute ischemic stroke treated with endovascular thrombectomy under general anesthesia. The mRS is a categorical scale ranging from 0 (no symptoms) to 6 (death), and it will be assessed by trained research staff blinded to the randomization. Success will be considered as an mRS score of 0–2.

#### Secondary objectives

The study will also investigate the effect of early (< 6 h) vs delayed (6–12 h) extubation after endovascular thrombectomy under general anesthesia for acute ischemic stroke, examining additional outcomes: the percentage of patients unable to be extubated in the assigned group, NIHSS scores on days 1 and 2, time to hospital discharge, mRS scores at hospital discharge, complications during ICU and hospital stay, and lengths of stay in the ICU and Hospital. We aim to identify factors that may influence poorer functional status at hospital discharge (NIHSS and mRS scores) and at 90 days (mRS score).

### Trial design {8}

The EDESTROKE trial is an investigator-initiated, single-center, prospective, parallel, evaluated blinded, superiority, randomized controlled trial.

#### CONSORT diagram

**Fig. 1:**
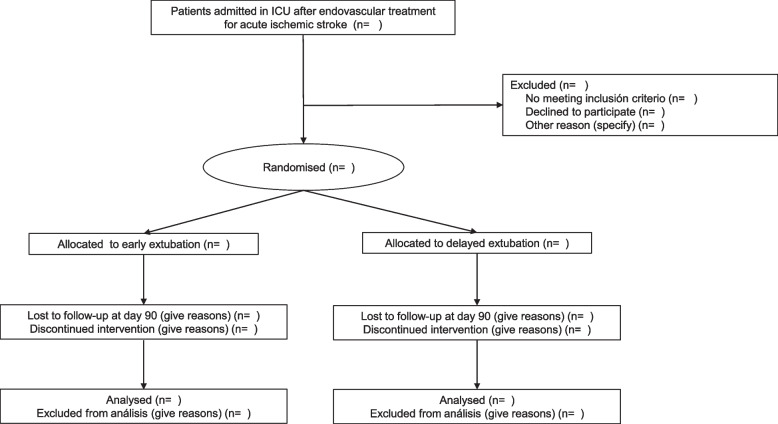
The Consolidated Standards of Reporting Trials (CONSORT) diagram of the EDESTROKE trial

Figure 1 shows the Consolidated Standards of Reporting Trials (CONSORT) diagram of the EDESTROKE trial.

## Methods: participants, interventions, and outcomes

### Study setting {9}

The EDESTROKE trial will be conducted in one university Hospital in Spain (University Clinical Hospital of Santiago de Compostela, Galicia, Spain).

### Eligibility criteria {10}

#### Inclusion criteria

- Adult (age ≥ 18 years), both genders.
- Acute ischemic stroke due to large intracranial vessel occlusion demonstrated on CT-angiography in the following anterior circulation locations (occlusion of the internal carotid artery and/or middle cerebral artery in segments M1, M2, M3) within 24 h of symptom onset.
- Patients admitted with a NIHSS neurological status ≥ 6.
- Patients who received endovascular treatment under general anesthesia and mechanical ventilation with satisfactory reperfusion (TICI 2b-2c-3).
- Patients intubated in the interventional radiology room.
- Patients admitted to the ICU with mechanical ventilation.
- Written informed consent from the patient or proxy (if present) before inclusion or once possible when patient has been included in a context of emergency.

#### Exclusion criteria

- Patients who have not been tracheal intubated in the interventional radiology room.
- Female subjects with childbearing potential who are pregnant at the time of screening
- Patients who suffer bronchial aspiration prior to the endovascular procedure or during tracheal intubation.
- Patients with pre-existing pulmonary condition potentially requiring ventilatory support.
- Patients who did not undergo the endovascular procedure under an anesthetic method other than general anesthesia with mechanical ventilation.
- Patients with functional neurological status, prior to the ischemic stroke, measured with the modified Rankin scale (mRS) of value: 3–6.
- Patients with vascular involvement of the posterior cerebral circulation, or intracranial hemorrhage associated with stroke.
- Patients who do not sign the informed consent by themselves or their relatives.

### Who will take informed consent? {26a}

Trial investigators will identify consecutive patients admitted to the ICU with acute ischemic stroke due to a proximal occlusion of the anterior circulation, who meet the inclusion criteria. Eligible patients or their legal representatives if the patient cannot understand information due to an underlying disease will receive written and oral information and will be included in randomization after investigators have obtained informed written consent. Once patients are extubated and if their medical condition allows them to understand the information, they will be given the option to either continue participating in the study or withdraw themselves from it.

### Additional consent provisions for collection and use of participant data and biological specimens {26b}

Not applicable. This trial does not involve collecting biological specimens for storage.

## Interventions

### Explanation for the choice of comparators {6b}

Patients with acute ischemic stroke due to a proximal occlusion of the anterior circulation admitted in ICU after successful endovascular thrombectomy under general anesthesia will be allocated to one of the following two study groups:

- Early extubation group: patients are extubated < 6 h after endovascular thrombectomy under general anesthesia.
- Delayed extubation group: patients are extubated 6–12 h after endovascular thrombectomy under general anesthesia.

### Intervention description {11a}

In both groups of patients undergoing endovascular thrombectomy in the interventional radiology room, general anesthesia will be administered at the discretion of the attending anesthesiologist. All patients will be monitored with electrocardiogram, hear rate, pulse oxygen saturation, invasive blood pressure, end-tidal carbon dioxide, inspired oxygen fraction, and blood glucose. Plasma glucose will be maintained within the range of 140–180 mg/dL, and systolic blood pressure will be targeted to be kept between 140 and 180 mmHg, with vasopressor support if necessary. Following endovascular treatment, patients will be transferred to the ICU, and if they meet the inclusion criteria, they will be then randomized into one of two groups: early vs delayed extubation.

In the ICU, patients will be monitored similarly to the interventional radiology room. Because the trial includes patients who have undergone satisfactory endovascular thrombectomy (TICI 2b-3), systolic blood pressure will be targeted to be maintained between 120 and 140 mmHg, with vasopressor support if necessary. Patients allocated to the early extubation group will be extubated within 6 h after endovascular thrombectomy, while those in the delayed extubation group will be extubated between 6 and 12 h after treatment.

During both assigned time frames, patients will be extubated if they meet the following criteria:

1. Hemodynamic stability: The patient should be hemodynamically stable, with blood pressure within acceptable ranges and no significant need for vasopressor support.
2. Respiratory stability: The patient should have adequate respiratory function, with effective mechanical ventilation and no signs of acute respiratory distress.
3. Neurological status: The patient should be awake, alert, and able to follow simple commands. Assessment of airway protection and swallowing capacity will be considered.
4. Assessment of cough and secretion clearance: The patient should have the ability to cough and clear secretions effectively to prevent respiratory complications.
5. Response to spontaneous ventilation: A spontaneous breathing trial may be conducted to assess the patient’s ability to breathe independently without ventilatory support.

If the patient cannot be extubated within assigned time frame for any reason, it will be documented. After extubation, if the patient is clinically stable, they will be transferred to the neurology hospitalization floor and subsequently discharged from the hospital. Ninety days after the endovascular thrombectomy, patients will have a consultation with a neurologist.

### Criteria for discontinuing or modifying allocated interventions {11b}

Patients and their authorized members can withdraw from the study at any time without any negative consequences attached. The study investigators reserve the right to temporarily or permanently halt a patient’s participation in the study if it is deemed to be in the patient’s best interest, particularly in response to a serious adverse event. Affected patients will no longer be subject to protocol monitoring but will continue to receive optimal care based on their health status and current knowledge. In the event of early study withdrawals, the investigator is required to thoroughly document the reasons. Premature termination of the study may occur if unexpected adverse events arise or if unforeseen circumstances or new medical information necessitate sponsor intervention. Any modifications to the study protocol must be documented by the principal investigator. Even in cases of protocol deviation, participant follow-up must continue until the specified endpoint.

### Strategies to improve adherence to interventions {11c}

To improve adherence, patients and their authorized family members will receive comprehensive explanations about the intervention, potential risks, and other details from the recruitment team. They will also be informed about the importance of completing follow-up assessments. They can reach us whenever they have any question or need medical attention.

Except for the extubation timing (early versus delayed extubation), the rest of the treatment in both groups will be provided within the standard of care. Any protocol deviations will be recorded on the electronic case report form, and a clinical research assistant will ensure that all protocol deviations and adverse events are recorded in the database.

### Relevant concomitant care permitted or prohibited during the trial {11d}

There is no specific concomitant care administered nor prohibited during the trial.

### Provisions for post-trial care {30}

There is no anticipated harm or compensation for trial participation.

### Outcomes {12}

#### Primary outcome measure

The primary outcome of this study will be the functional outcome assessed with the modified Rankin Scale (mRS), 90 days after the endovascular thrombectomy. To improve the follow-up rate, we defined 90 days as 90 ± 14 days after the endovascular thrombectomy to consider situations such as holidays. The mRS is a 7-grade categorical scale ranging from 0 (no symptoms) to 6 (death). Success will be considered as a mRS of 0–2 (functional independence). The mRS will be assessed by trained research staff blinded to the randomization group.

#### Secondary outcomes measures

The secondary outcomes of this study will be as follows:

1. NIHSS score at day 1, day 2, and at the time of hospital discharge.
2. mRS at the time of hospital discharge.
3. Percentage of patients who cannot be extubated in the assigned group.
4. Complications associated with mechanical ventilation: pulmonary injuries (barotrauma or volutrauma), respiratory infections (ventilator-associated pneumonia, bronchoaspiration), airway, oral or pharyngeal injuries, and others.
5. Complications during ICU and hospital stay: intracranial hemorrhage, hemorrhagic transformation, reperfusion syndrome, need for craniectomy, prolonged mechanical ventilation in ICU (> 24 h), prolonged mechanical ventilation in ICU (> 48 h), reintubation, prolonged ICU stay (> 48 h), pneumonia, bronchoaspiration, other infection, in-ICU mortality, in-hospital mortality, and other complications.
6. ICU and Hospital length of stay.
7. Work-flow time, including stroke onset to door time, to artery puncture, and to reperfusion or procedure completion.

### Participant timeline {13}

The participant time lime is described in Table 1.

**Table 1:** Schedule of enrollment, intervention and assessment

Participant timeline	At arrival	During treatment	After treatment	1–12 h after treatment	24 h after treatment	48 h after treatment	Hospital discharge	3 months after treatment
**Enrolment**								
Eligibility assessment			**X**					
Informed consent			**X**					
Allocation			**X**					
**Interventions**								
Early extubation (< 6 h)				**X**				
Delayed extubation (6–12 h)				**X**				
**Assessments**								
Baseline variables	**X**							
Laboratory testing	**X**				**X**		**X**	
Brain image	**X**				**X**			
NIHSS score	**X**				**X**	**X**	**X**	
mRS score	**X**						**X**	**X**
TICI reperfusion		**X**						
Length of hospital and ICU stay							**X**	
Complications associated with VM		**X**	**X**	**X**	**X**	**X**	**X**	
Complications during hospital stay				**X**	**X**	**X**	**X**	

*ICU* intensive care unit, *mRS* modified Rankin Scale (ranges from 0 (no symptom) to 6 (death)), *NIHSS score* National Institutes of Health Stroke scale (range 0–42, higher scores indicate more severe neurologic deficits), *TICI reperfusion* thrombolysis in cerebral infarction, TICI 2b-2c-3 indicate successful reperfusion, *MV* mechanical ventilation

### Sample size {14}

We aim to compare neurological functional status at 90 days according to the modified Rankin scale (mRS), of patients with stroke who underwent endovascular thrombectomy with satisfactory results and who underwent early extubation (< 6 h) compared to delayed extubation (6–12 h). Efficacy will be assessed with the proportion of patients with functional independence (mRS scale ≤ 2).

A previous observational study [20] reported that 55.5% of patients achieved an mRS score of ≤ 2 after receiving early extubation (mechanical ventilation for less than 6 h), compared to 33.9% when extubation was delayed (mechanical ventilation for more than 6 h). In present trial, we hypothesize that early extubation, compared with delayed extubation, is associated with a better functional outcome 3 months after endovascular thrombectomy under general anesthesia for acute ischemic stroke. We have decided to set the level of significance at 5% (*α* = 0.05), with a power of 80% (*β* = 0.80), and to use a bilateral test (c = 2). We will work with two independent samples and both groups will be balanced (w1 = 0.5). Considering an expected percentage of losses is 5.00%, it would be necessary to include 87 individuals in each group, totaling 174 patients in the study. Depending on the course of the study, an interim analysis will be conducted when 70% of the recruited sample is reached, if deemed appropriate by the research team.

- We hypothesized that early compared with delayed extubation is associated with a better functional outcome 3 months after endovascular thrombectomy treatment under general anesthesia for acute ischemic stroke.

### Recruitment {15}

Patients admitted to the ICU with acute ischemic stroke due to a proximal occlusion of the anterior circulation who meet the inclusion criteria will be recruited by trial investigators. Eligible patients, or their legal representatives if the patient cannot understand information due to an underlying disease, will receive both written and oral information. They will be included in randomization after investigators have obtained informed written consent. Patients are expected to be enrolled over a 3-year period starting in April 2023. To date (February 2024), 70 patients have been randomized in the trial.

## Assignment of interventions: allocation

### Sequence generation {16a}, concealment mechanism {16b}, implementation {16c}

Randomization will be performed immediately after the patient is admitted to the ICU. Enrolled participants will be randomized in a 1:1 ratio for early or delayed extubation. To ensure balance between the two groups, a computer-generated random block number table will be used (blocks of 8). A designated staff member, not involved in extubation, anesthesia management, or follow-up will handle recruitment and generate the allocation randomization sequence. The staff member will implement the allocation sequence through opaque, sealed, and stapled envelopes.

## Assignment of interventions: blinding

### Who will be blinded {17a}, procedure for unblinding if needed {17b}

The primary outcome (mRS assessed 90 days after the endovascular thrombectomy) and secondary outcomes such as NIHSS at day 1, day 2, and at hospital discharge, as well as mRS at hospital discharge, complications during Hospital stay outside the ICU, and hospital length of stay, will be assessed by a neurologist from the trial team who is blinded to the randomization group. Complications during ICU stay, duration of mechanical ventilation, and complications associated with mechanical ventilation will be assessed by a trial team staff member who is not blinded.

## Data collection and management

### Plans for assessment and collection of outcomes {18a}, plans to promote participant retention and complete follow-up {18b}, data management {19}, confidentiality {27}

Pseudonymized data will be collected and managed in an electronic case report form (eCRF) specifically designed and validated for this purpose. Access to data will comply with local, national, and European Union data protection regulations. Each patient will be assigned a unique trial identification number, which will be disassociated from their personal information. The database will be protected by encryption software. Only the investigators and research team will have access to any protected health information of study participants and any study data. Any protocol deviations will be recorded on the eCRF, and a clinical research assistant will ensure that all protocol deviations and adverse events are recorded in the database. The following data will be registered:

Baseline characteristics and intraoperative data will include:

- Demographic data (age, height, weight, gender, and body mass index)
- American Society of Anesthesiologists physical status
- Significant comorbidities (cardiovascular, respiratory, neurological, cancer, atrial fibrillation, smoking, statin use, antiplatelet and anticoagulant treatments)
- NIHSS score at admission
- Time of symptom onset, time of arrival at the hospital, time of first image for diagnosis, time of arterial puncture, time of tracheal intubation, time of recanalization
- Type of anesthesia (general anesthesia vs. sedation)
- Modified Thrombolysis in Cerebral Infarction (mTICI) score
- Need for norepinephrine during endovascular treatment
- Intraoperative complications (hypotension, hypertension, duration of procedure, stroke localization, intravenous fibrinolysis if applicable)
- Type of endovascular stroke treatment (direct aspiration, stent retriever, angioplasty, etc.)
- Procedural-related complications.

ICU data:

- Duration of mechanical ventilation (hours)
- pO2 at ICU admission
- Time of extubation
- Need for norepinephrine during mechanical ventilation and after mechanical ventilation
- Mechanical ventilation complications (pneumothorax, pneumonia, etc.)
- Duration of ICU stay (hours)
- Complications during ICU stay (reintubation, infection, prolonged mechanical ventilation (> 48 h), prolonged ICU stay (> 48 h), ICU mortality, and other complications)

Hospital data:

- NIHSS score on day 1, day 2, and at hospital discharge
- mRS at hospital discharge and 3 months after endovascular treatment
- Complications during hospital stay
- Duration of hospital stay (days)

#### Access to data

Data safety, data quality, and statistical analysis will be managed by two investigators (MT, AEG), who are responsible for notifying any issues that may arise during the whole study. Data will be collected and stored according to good clinical practice guidelines and will be available to all participants. Any issues occurring during the clinical trial will be reported to these two investigators.

### Plans for collection, laboratory evaluation, and storage of biological specimens for genetic or molecular analysis in this trial/future use {33}

Not applicable as no biological samples will be collected for future analysis.

## Statistical methods

### Statistical methods for primary and secondary outcomes {20a}

#### Description of the patient groups at baseline

The baseline characteristics of all patients will be described. Categorical variables will be reported as absolute numbers (*n*) and percentages and quantitative variables as either means (standard deviation) or medians (25th percentile–75th percentile). Data will be analyzed using chi-square and Wilcoxon rank-sum test (or paired samples *T* test) as appropriate. Pearson or Spearman correlation coefficient will be calculated to compare patient specifics between groups at the baseline. Normality will be calculated with Kolmogorov–Smirnov test with Lilliefors correction.

#### Analysis of the primary outcome

To answer the primary outcome, functional independence assessed with the modified Rankin Scale (mRS) will be evaluated 90 days after the endovascular thrombectomy by trained research staff who are blinded to the randomization group. To enhance the follow-up rate, we have defined the assessment window as 90 ± 14 days after the procedure to account for factors such as holidays. The mRS is a 7-grade categorical scale ranging from 0 (no symptoms) to 6 (death). A dichotomous variable “efficacy” (success) will be created from the mRS scale, with success defined as an mRS of 0–2 indicating functional independence.

To determine the efficacy between the two clinical approaches, regression techniques will be employed, and the relative risk along with its 95% confidence (RR [IC 95%]) will be calculated. Univariate logistic regression models will be built for each independent variable. Selection of independent variables for inclusion in multivariate models will be based on clinical judgment and statistical considerations, including correlation with the dependent variable, and the coefficients and *p*-values obtained in the univariate models. Multivariate models with the selected independent variables candidates will be built using stepwise criteria, and the Akaike Information Criterion (AIC) will be calculated to identify the model that best explains the outcome.

#### Analysis of the secondary outcomes

The techniques that will be used to answer the secondary objectives will be fundamentally statistical inference techniques. The selection of each of them will depend on the nature of the variables involved, as well as their adjustment to normality.

All these analyses will be carried out with the statistical program SPSS 19.0 and those whose *p*-value is lower than 0.05 will be considered statistically significant values.

### Interim analyses {21b}

A safety interim analysis is planned after 70% of the recruited sample is reached, if the research team deems it appropriate.

### Methods for additional analyses (e.g., subgroup analyses) {20b}

Primary outcome will be analyzed in following subgroups: age, gender, body mass index, NIHSS score at admission, ASPECTS, site of arterial occlusion, time from symptom onset to endovascular thrombectomy, use of intravenous thrombolysis.

### Methods in analysis to handle protocol non-adherence and any statistical methods to handle missing data {20c}

We will conduct analyses based on the intention-to-treat principle, meaning that participants are included in the final analysis regardless of adherence to the assigned treatment. As a sensitivity analysis, per-protocol analysis may be employed to complement the main analysis results. Missing data will be addressed through inverse probability weighting and worst-case imputation methods.

### Plans to give access to the full protocol, participant-level data, and statistical code {31c}

#### Plan to share

Data types: Deidentified participant data.

How to access data: Requests must be sent to *manutabo@yahoo.es.*

When available: With publication.

Who can access the data: Researchers whose proposed use of the data has been approved.

Types of analyses: For scientific purpose.

Mechanisms of data availability: With investigator support.

## Oversight and monitoring

### Composition of the coordinating center and trial steering committee {5d}

#### Composition of the data monitoring committee, its role and reporting structure {21a}

Before the start of the study, both anesthetic and neurological medical teams will undergo formal training sessions on the study protocol and data collection procedures using the CRF. All necessary documents for the study will be readily available to each researcher. Medical researchers will oversee patients screening and inclusion while data will be collected in a web-based eCRF by trial personal. Each patient will be assigned a unique trial identification number, with only the investigators and research team will have access to any protected health information or study data.

The trial’s day-to-day coordination will be managed by two anesthesiologists (MT and JF) and one neurologist (MRY) from the University Clinical Hospital of Santiago, along with support from an epidemiologist (AEG) from the Sanitary Research Institute of Santiago of Compostela.

There is no data and safety monitoring board because of the low expectance of safety concerns or risk for participants. We ensure ethical oversight throughout the study conduct to safeguard the rights, safety, and well-being of all participants involved. This includes adherence to established ethical principles, compliance with relevant regulations and guidelines, and obtaining approval from appropriate ethics committees or institutional review boards.

### Adverse event reporting and harms {22}

The study may be temporarily halted for an individual patient, at the discretion of the attending physician, in case of major serious adverse events suspected to be associated with the assigned mechanical ventilation time (early extubation (< 6 h) or delayed extubation (6–12 h)). No specific reporting procedure for unexpected serious adverse events is planned because strategies used in the two periods studied are standard care in our hospital.

### Frequency and plans for auditing trial conduct {23}

The Research Methodology Unit, *Fundación Instituto de Investigaciones Sanitarias* (FIDIS) of Santiago, will review the screening forms and clinical data at regular intervals.

### Plans for communicating important protocol amendments to relevant parties (e.g., trial participants, ethical committees) {25}

The Santiago-Lugo Research Ethics Committee (CEI-SL) will be informed for any new protocol amendments and will be asked for approval.

### Dissemination plans {31a}

Trial results will be submitted to a peer-reviewed journal and will be presented at one or more scientific conferences.

## Discussion

To the best of our knowledge, the EDESTROKE trial is the first randomized controlled study comparing the effect of mechanical ventilation duration (early vs delayed extubation) following satisfactory endovascular thrombectomy for acute ischemic stroke under general anesthesia.

Two previous studies have demonstrated that longer mechanical ventilation times were associated with a higher incidence of pneumonia and a worse functional outcome at 3 months [20, 21]. However, these two studies were observational, and several factors may have influenced the results. Factors such as age, pre-stroke mRS score, size and localization of the cerebral infarct, time between the symptoms onset of recanalization, adequate control of blood pressure during and after the endovascular thrombectomy, NIHSS score or neurological status at the time of the procedure, and successful recanalization (TICI 2b-3) may influence the outcomes of patients who have suffered a stroke and received endovascular thrombectomy, thereby potentially affecting the success of extubation after the procedure under general anesthesia [16, 17].

Currently, there are no guidelines recommending the optimal moment of extubation following endovascular thrombectomy under general anesthesia for stroke patients. Prolonged mechanical ventilation times may be associated with an increase in complications such as pneumonia or cerebral blood flow disturbances, due to the vasodilatation produced by anesthetic drugs. Early extubation in stroke patients may lead to complications such as agitation, disorientation, loss of reflexes, sudden fluctuations in blood pressure, alterations in cerebral blood flow, respiratory distress, bronchial aspiration, and the need for reintubation [16–19].

In the current study, we aim to compare two different periods of extubation following satisfactory endovascular treatment for acute stroke: extubation within < 6 h of mechanical ventilation (early extubation) versus extubation within 6–12 h of mechanical ventilation (delayed extubation). To minimize potential confounding factors, we have decided to include only patients meeting specific criteria in both study groups. These criteria include acute ischemic stroke of the anterior circulation, NIHSS neurological status ≤ 6, successful recanalization (TICI 2b-3), intubation performed by an anesthesiologist in the interventional radiology room, absence of bronchial aspiration prior to or during intubation, pre-stroke mRS ≤ 2, and adequate control of blood pressure during and after the procedure.

After the study, we hope to have gathered sufficient data to recommend the optimal timing for extubation following endovascular thrombectomy under general anesthesia for patients with acute ischemic stroke. The findings of the study would serve as a reference for future trials investigating the effects of optimal extubation timing in patients with acute ischemic stroke of the posterior circulation or those with unsatisfactory recanalization.

## Trial status

Protocol version 1; April 2023.

The trial started and actively enrolling since April 18, 2023**.**

Primary completion: September 30, 2026 (approximate date when recruitment will be completed).

### Supplementary Information

Supplementary Material 1.

## Data Availability

Pseudonymized data will be collected and managed in an electronic case report form (eCRF) designed specifically for this purpose. Each patient will receive a unique trial identification number, disassociated from their personal information. The database will be protected by encryption software and a single user password will be provided to each anesthesiologist or neurologist. Only the investigators and research team will have access to any protected health information of study participants and any study data. Any protocol deviations will be recorded on the eCRF, and a clinical research assistant ensure that all protocol deviations and adverse events will be recorded in the database. Data safety, data quality, and statistical analysis will be managed by two investigators (MT, AEG), who are responsible for notifying any issues that may arise during the whole study. Data will be collected and stored according to good clinical practice guidelines and will be available to all participants. Any issue occurring during the clinical trial will be reported to these two investigators.

## Abbreviations

ICU: Intensive care unit
mRS: Modified Rankin Scale (ranges from 0 (no symptom) to 6 (death))
NIHSS score: National Institutes of Health Stroke scale (range 0–42, higher scores indicate more severe neurologic deficits)
TICI reperfusion: Thrombolysis in cerebral infarction, TICI 2b-2c-3 indicate successful reperfusion
MV: Mechanical ventilation
