# Supplementary material for: Effect of early vs. delayed extubation on functional outcome among patients with acute ischemic stroke treated with endovascular thrombectomy under general anesthesia: the prospective, randomized controlled EDESTROKE trial study protocol
Source: Trials. 2024 Jun 4;25:357. doi: 10.1186/s13063-024-08181-y (PMC11151624; doi:10.1186/s13063-024-08181-y)
Supplement: Supplementary file 1 — Supplementary Material 1. [file 13063_2024_8181_MOESM1_ESM.pdf]

<u>HOJA DE INFORMACIÓN AL/LA PARTICIPANTE ADULTO/A:</u>

<u>TÍTULO DEL ESTUDIO</u>

Impacto del tiempo de ventilación mecánica en el estado neurológico, tras tratamiento endovascular bajo anestesia general en pacientes con ictus isquémico agudo.

Estudio prospectivo, de intervención, randomizado, ciego para el evaluador y de grupos paralelos comparando una extubación temprana (<6 horas) o retardada (6-12 horas).

INVESTIGADOR: Manuel Taboada Muñiz, Investigador principal
CENTRO: Complexo Hospitalario Santiago, Área Sanitaria de Santiago de Compostela e Barbanza.

Este documento tiene por objeto ofrecerle información sobre un **estudio de investigación** en el que se le invita a participar. Este estudio fue aprobado por el Comité de Ética de la Investigación de Galicia.

Si decide participar en el mismo, debe recibir información personalizada del investigador, **leer antes este documento** y hacer todas las preguntas que precise para comprender los detalles sobre el mismo. Si así lo desea puede consultarlo con otras personas y tomar el tiempo necesario para decidir si participa o no.

La participación en este estudio es completamente **voluntaria**. Ud. puede decidir no participar o, se acepta hacerlo, cambiar de parecer retirando el consentimiento en cualquier momento sin dar explicaciones. Le aseguramos que esta decisión no afectará a la relación con los profesionales sanitarios que le atienden ni a la asistencia sanitaria a la que Ud. tiene derecho.

¿Cuál es la finalidad del estudio?

El objetivo del estudio consiste en comparar dos tiempos de extubación utilizados habitualmente: "**extubación temprana (<6 horas) o retardada (6-12 horas)"** tras tratamiento endovascular bajo anestesia general en pacientes con ictus isquémico agudo.

¿Por qué me ofrecen participar a mí?

Ud. está invitado a participar porque va a recibir o ha recibido una anestesia general para realizar un tratamiento endovascular por padecer un ictus isquémico agudo. Tras el procedimiento, se le va a despertar y proceder a extubación. Nos gustaría recoger una serie de datos relacionados con el momento de la extubación (extubación temprana o retardada), que son dos métodos que utilizamos habitualmente en nuestro Hospital para extubación tras este procedimiento.

¿En qué consiste mi participación?

Su participación no supone ninguna variación en el manejo del paciente sometido a tratamiento endovascular por ictus isquémico agudo, salvo que la decisión de extubar de forma temprana (primeras 6 horas tras el procedimiento) o de forma retardada (entre 6 y 12 horas tras el procedimiento), que habitualmente se usan de forma indistinta, va a depender del azar. Es decir, que el procedimiento de asignación al grupo de "extubación temprana" o el "extubación retardada", es un proceso aleatorio, porque está demostrado que es la forma idónea para conseguir grupos de pacientes comparables.

A la mitad de los pacientes reclutados, se les realizará “extubación temprana” y a la otra mitad “extubación retardada”. Por tanto, tiene una probabilidad del 50% de que se le realice uno de los dos procedimientos. Usted no sabrá qué técnica utilizaremos con usted.

Su participación consiste, además, en permitirnos acceder a su historia clínica, para obtener una serie de datos clínicos necesarios para el estudio.

¿Qué molestias o inconvenientes tiene mi participación?

Su participación no implica molestias adicionales a las de la práctica asistencial habitual.

¿Obtendré algún beneficio por participar?

No se espera que Ud. obtenga beneficio directo por participar en el estudio. La investigación pretende comparar aspectos sobre el uso de **dos métodos diferentes de extubación usados habitualmente en la práctica clínica habitual** tras tratamiento endovascular bajo anestesia general del ictus isquémico agudo.

Esta información podrá ser de utilidad en un futuro para otras personas.

¿Recibiré la información que se obtenga del estudio?

Si Ud. lo desea, se le facilitará un resumen de los resultados del estudio.

¿Se publicarán los resultados de este estudio?

Los resultados de este estudio serán remitidos a publicaciones científicas para su difusión, pero no se transmitirá ningún dato que permita la identificación de los participantes.

Información referente a sus datos:

La obtención, tratamiento, conservación, comunicación y cesión de sus datos se hará conforme a lo dispuesto en el Reglamento General de Protección de Datos (Reglamento UE 2016-679 del Parlamento europeo y del Consejo, de 27 de abril de 2016) y la normativa española sobre protección de datos de carácter personal vigente.

La institución en la que se desarrolla esta investigación es la responsable del tratamiento de sus datos pudiendo contactar con el Delegado/a de Protección de Datos a través de los siguientes medios: delegado.proteccion.datos@sergas.es

Los datos necesarios para llevar a cabo este estudio serán recogidos y conservados de modo **Seudonimizados (Codificados):** la seudonimización es el tratamiento de datos personales de manera tal que no pueden atribuirse a un/a interesado/a sin que se use información adicional. En este estudo solamente el equipo investigador conocerá el código que permitirá saber su identidad.

La normativa que regula el tratamiento de datos de personas le otorga el derecho a acceder a sus datos, oponerse, corregirlos, cancelarlos, limitar su tratamiento, restringir o solicitar la supresión de los mismos. También puede solicitar una copia de éstos o que ésta sea remitida a un tercero (derecho de portabilidad).

Para ejercer estos derechos puede Ud. dirigirse al Delegado/a de Protección de Datos del centro a través de los medios de contacto antes indicados o al investigador/a principal de este estudio en el correo electrónico: manutabo@yahoo.es, y/o 981950674.

Así mismo, Ud. tiene derecho a interponer una reclamación ante la Agencia Española de Protección de datos cuando considere que alguno de sus derechos no haya sido respetado.

Únicamente el equipo investigador y las autoridades sanitarias, que tienen el deber de guardar la confidencialidad, tendrán acceso a todos los datos recogidos por el estudio. Se podrá transmitir a terceros información que no pueda ser identificada. En el caso de que alguna información se transmita a otros países, se realizará con un nivel de protección de datos equivalente, como mínimo, al establecido por la normativa española y europea.

Al finalizar el estudio, o el plazo legal establecido, los datos recogidos serán eliminados o guardados anónimos para su uso en futuras investigaciones según lo que Ud. escoja en la hoja de firma del consentimiento

¿Existen intereses económicos en este estudio?

Esta investigación es promovida por el investigador principal sin fondos específicos para realizarlo.

El investigador no recibirá retribución específica por la dedicación al estudio.

Ud. no será retribuido por participar. Es posible que de los resultados del estudio se deriven productos comerciales o patentes; en este caso, Ud. no participará de los beneficios económicos originados.

¿Cómo contactar con el equipo investigador de este estudio?

Ud. puede contactar con MANUEL TABOADA MUÑIZ en el teléfono 981950674 y/o el correo electrónico: manutabo@yahoo.es, y manuel.taboada.muniz@sergas.es

Muchas gracias por su colaboración

DOCUMENTO DE CONSENTIMIENTO DEL PACIENTE ADULTO PARA LA PARTICIPACIÓN EN UN ESTUDIO DE INVESTIGACIÓN

TÍTULO del estudio:

Impacto del tiempo de ventilación mecánica en el estado neurológico, tras tratamiento endovascular bajo anestesia general en pacientes con ictus isquémico agudo.

Estudio prospectivo, de intervención, randomizado, ciego para el evaluador y de grupos paralelos comparando una extubación temprana (<6 horas) o retardada (6-12 horas).

Yo,…………………………………………………………………………………………………

- Leí la hoja de información al participante del estudio arriba mencionado que se me entregó, pude conversar con ………………………………………………(investigador del estudio)
- y hacer todas las preguntas sobre el estudio.
- Comprendo que mi participación es voluntaria, y que puedo retirarme del estudio cuando quiera, sin tener que dar explicaciones y sin que esto repercuta en mis cuidados médicos.
- Accedo a que se utilicen mis datos en las condiciones detalladas en la hoja de información al participante.
- Presto libremente mi conformidad para participar en este estudio.

Al terminar este estudio acepto que mis datos sean:

☐ Eliminados

☐ Conservados anonimizados para usos futuros en otras investigaciones

Fdo.: El/la participante,

Fdo.: El/la investigador/a que solicita el consentimiento

Nombre y Apellidos:

Nombre y Apellidos:

Fecha:

Fecha:

Se deberán firmar dos modelos, uno será entregado al participante y otro será conservado por el responsable del estudio de investigación

DOCUMENTO DE CONSENTIMIENTO ANTE TESTIGOS PARA LA PARTICIPACIÓN EN UN ESTUDIO DE INVESTIGACIÓN (para los casos en que el participante no pueda leer/escribir)

El testigo imparcial ha de identificarse y ser una persona ajena al equipo investigador.

TÍTULO del estudio:

Impacto del tiempo de ventilación mecánica en el estado neurológico, tras tratamiento endovascular bajo anestesia general en pacientes con ictus isquémico agudo.

Estudio prospectivo, de intervención, randomizado, ciego para el evaluador y de grupos paralelos comparando una extubación temprana (<6 horas) o retardada (6-12 horas).

Yo,………………………………………………………., como testigo imparcial, afirmo que en mi presencia:

- Se le leyó a………………………………… la hoja de información al participante del estudio arriba mencionado que se le entregó, y pudo hacer todas las preguntas sobre el estudio.
- Comprendió que su participación es voluntaria, y que puede retirarse del estudio cuando quiera, sin tener que dar explicaciones y sin que esto repercuta en sus cuidados médicos.
- Accede a que se utilicen sus datos en las condiciones detalladas en la hoja de información al participante.
- Presta libremente su conformidad para participar en este estudio.

Al terminar este estudio acepta que sus datos sean:

☐ Eliminados

☐ Conservados anonimizados para usos futuros en otras investigaciones

Fdo.: El/la testigo,

Fdo.: El/la investigador/a que solicita el consentimiento

Nombre y apellidos:

Nombre y Apellidos:

Fecha:

Fecha:

REVOCACIÓN/AUTORIZACIÓN CONSENTIMIENTO INFORMADO

Mediante el presente documento declaro la

☐ REVOCACIÓN

☐ AUTORIZACIÓN

del consentimiento informado firmado el día .... en el que se consintió mi participación en el estudio “Impacto del tiempo de ventilación mecánica en el estado neurológico, tras tratamiento endovascular bajo anestesia general en pacientes con ictus isquémico agudo”

Nombre Paciente:...

Fecha:...

Firma Paciente:

DOCUMENTO DE CONSENTIMIENTO PARA REPRESENTANTE LEGAL PARA LA PARTICIPACIÓN EN UN ESTUDIO DE INVESTIGACIÓN

TÍTULO del estudio:

Impacto del tiempo de ventilación mecánica en el estado neurológico, tras tratamiento endovascular bajo anestesia general en pacientes con ictus isquémico agudo.

Estudio prospectivo, de intervención, randomizado, ciego para el evaluador y de grupos paralelos comparando una extubación temprana (<6 horas) o retardada (6-12 horas).

Yo, ____, representante legal de ____

- Leí la hoja de información al participante del estudio arriba mencionado que se me entregó, pude conversar con ………………………. y hacer todas las preguntas sobre el estudio.
- Comprendo que su participación es voluntaria, y que puede retirarse del estudio cuando quiera, sin tener que dar explicaciones y sin que esto repercuta en sus cuidados médicos.
- Accedo a que se utilicen sus datos en las condiciones detalladas en la hoja de información al participante.
- Presto libremente mi conformidad para que participe en este estudio.

Al terminar este estudio acepto que sus datos sean:

☐ Eliminados

☐ Conservados anonimizados para usos futuros en otras investigaciones

Fdo.: El/la representante legal,

Nombre e apellidos:

Fecha:

Fdo.: El/la investigador/a que solicita el consentimiento

Nombre e apellidos:

Fecha:

Versión: 1.0 data 02 de marzo de 2023

Se deberán firmar dos modelos, uno será entregado al participante y otro será conservado por el responsable del estudio de investigación
